# Comparison of mandibular cross-sectional morphology between Class I and Class II subjects with different vertical patterns: based on CBCT images and statistical shape analysis

**DOI:** 10.1186/s12903-021-01591-3

**Published:** 2021-05-05

**Authors:** Haotian Chen, Zijin Liu, Xinnong Hu, Ben Wu, Yan Gu

**Affiliations:** 1 Department of Orthodontics, National Engineering Laboratory for Digital and Material Technology of Stomatology, Beijing Key Laboratory of Digital Stomatology, Peking University School and Hospital of Stomatology, No. 22 Zhongguancun Avenue South, Haidian District, Beijing, 100081 China; 2 Center for Applied Statistics, School of Statistics, Renmin University of China, No. 59 Zhongguancun Street, Haidian District, Beijing, 100872 China

**Keywords:** Mandibular morphology, Geometric morphometric measurement, Vertical pattern, Skeletal Class II

## Abstract

**Background:**

This study is aimed to (1) investigate the influence of sagittal and vertical patterns on mandibular cross-sectional morphology and to (2) provide visualized mandibular cross-sectional morphology in different groups with General Procrustes Analysis (GPA), canonical variance analysis (CVA) and discriminant function analysis (DFA).

**Methods:**

324 cone-beam computed tomography (CBCT) images were collected to analyze mandibular cross-sectional morphology and were categorized into 12 groups according to sagittal and vertical pattern and gender. One-way analysis of variance (ANOVA) was used to compare the difference among the groups. Thirty equidistant points were marked along the contour of mandibular cross-section and GPA, CVA and DFA were applied.

**Results:**

(1) Mandibular height in hyperdivergent groups was significantly higher than that in normodivergent and hypodivergent groups (*P* < 0.05). (2) Hypodivergent groups showed significantly wider upper third of mandibular width from symphysis to molar region than that in hyperdivergent group (*P* < 0.05), except for the premolar and molar regions in male groups (*P* > 0.05). (3) Class II hyperdivergent group showed narrowest lower third width in the molar region, with the mean value of 12.03 mm in females and 11.98 mm in males. (4) For males and females, the ratio between height and lower third width at symphysis was significantly higher in Class II hyperdivergent group than that in Class I hyperdivergent group (*P* < 0.05).

**Conclusions:**

(1) The influence of vertical facial patterns on mandibular cross-sectional morphology is more obvious than that of sagittal skeletal pattern. (2) Subjects with increased vertical dimension presented with a remarkable “slimer” mandibular cross-sectional morphology at symphysis. (3) A deeper curve along the anterior contour of symphysis in Class II hyperdivergent group was noted with GPA.

**Supplementary Information:**

The online version contains supplementary material available at 10.1186/s12903-021-01591-3.

## Background

Prediction of the possibility of periodontal damage due to orthodontic tooth movement plays an important role in treatment planning. Dental compensation of mild or moderate skeletal discrepancy with orthodontic treatment will result in change in inclination of teeth. Due to the fact that cone beam computed tomography (CBCT) should not be taken routinely for the orthodontic patients [1], comprehensive assessment of mandibular cross-sectional morphology and bone limitation is very important to estimate or minimize the risks of iatrogenic damage, including dehiscence and fenestration during the tooth movement.

CBCT images have been used in mandibular morphometric studies. Swasty et al. [2] used CBCT to investigate the mandibular cortical thickness, height and width and determined their relationship with age. However, majorities of studies focused on the influence of vertical pattern on alveolar thickness of symphysis or in molar area [3–7]. Gracco et al. [8] described mandibular incisor bony support using CBCT and concluded that long face group had slimmer symphysis than low face group. Sadek et al. [5] investigated the relationship between facial type and posterior alveolar thickness in both maxilla and mandible based on CBCT images and concluded high angle group presented thinner alveolus anteriorly in the maxilla and at almost all sites in the mandible. In a previous study, Baysal et al. found labial alveolar bone thickness was significantly thicker in Class I group compared to Class II group, thus the range of lower incisor movement in high-angle Class II patients is limited compared to average-angle Class II patients [9]. Therefore, it is reasonable to speculate that sagittal factors might potentially contribute to the variability of mandibular morphology. It will lead to corrected interpretation of the underlying characteristics among different facial pattern groups.

Statistical shape analysis, including general Procrustes analysis (GPA), principle component analysis (PCA), canonical variance analysis (CVA) and discriminant function analysis (DFA) methods have been increasingly used in morphometric studies [10–13]. GPA provides a metric to minimize in order to superimpose several shapes annotated by landmark points, which could compute the mean shape [14]. CVA is a widely used method for analyzing group structure in multivariate data and is mathematically equivalent to a one-way multivariate analysis of variance [15]. DFA performs a multivariate test of differences between groups and is used to determine the minimum number of dimensions needed to describe these differences [16]. Ahn et al. used PCA and structural equation modeling to reveal the correlation between facial skeletal pattern and mandibular morphology and concluded that vertical facial skeletal pattern was associated with the morphology of symphysis [10], while transverse facial skeletal pattern was associated with the morphology of mandibular cross-sections in the first molar region [11]. Zhang et al. [12] used GPA to reveal the alveolar bone change after retraction following premolar extraction. Bertl et al. [13] also used GPA to compare the difference of mandibular cross-section morphology between patients with second premolar agenesis and control group.

As mentioned above, previous studies could not depict different region of mandible, illustrate the morphology change and provide a whole picture of dynamic change of mandibular cross-sectional contour from symphysis to molar region. Therefore, the aim of this retrospective study includes: (1) to investigate the influence of sagittal and vertical patterns on mandibular cross-sectional morphology; (2) to provide visualized mandibular cross-sectional morphology in different groups with GPA, CVA and DFA.

## Methods

Three hundred and twenty-four CBCT images in this study were collected in Department of Oral and Maxillofacial Radiology, Peking University School and Hospital of Stomatology from May, 2014 to June, 2020, with mean age of 22.13 ± 2.78 years. The range of age is 18.00 to 25.83 years in males and 18.25 to 25.92 in females. This project was approved by Biomedical Ethics Committee of Peking University School and Hospital of Stomatology and the ethical number is PKUSSIRB-201951178.

### Inclusion and exclusion criteria

Inclusion and exclusion criteria were listed in Table 1.

**Table 1:** Inclusion and exclusion criteria of subjects

Inclusion criteria	Exclusion criteria
Mongolian	Skeletal Class III malocclusion
Skeletal Class I or Class II	Severe crowding
18.0–25.9 years old	Asymmetry in facial appearance
Permanent dentition	History of dental injuries
No head injury or other oral-maxillofacial diseases	
No congenitally missing, extracted or supernumerary teeth	
No orthodontic or orthognathic treatment	
No endodontic or periodontal diseases	
No systematic diseases	

The angle between mandibular plane (Gonion, Go-Gnathion, Gn) and Nasion-Sella line was defined as mandibular plane angle (MP). Hyperdivergent subjects were defined as MP > 37.7°, while normodivergent subjects were defined as 27.3° < MP ≤ 37.7°, and hypodivergent subjects were defined as MP ≤ 27.3° [4]. Skeletal Class I subjects were defined as 0.7° ≤ ANB < 4.7° and skeletal Class II subjects as ANB ≥ 4.7° and SNB ≤ 76.2° [17, 18].

The information of the subjects in each group was listed in Table 2. A minimum sample size of 14 subjects were required per group to achieve a significant analysis with significance level of 0.01 and statistical power of 90% by PASS software (Version 11, NCSS, Kaysvile, Utah) using symphysis height measurement in pilot study [19, 20].

**Table 2:** Information of subjects in each group

N (Mean age ± SD)	Female			Male		
	Class I	Class II	Total	Class I	Class II	Total
Hyperdivergent	33 (21.41 ± 2.63)	35 (21.75 ± 2.85)	68	22 (22.16 ± 3.06)	24 (22.04 ± 2.40)	46
Normodivergent	37 (22.56 ± 2.46)	31 (22.14 ± 2.86)	68	30 (21.04 ± 2.63)	27 (21.05 ± 2.45)	57
Hypodivergent	32 (22.74 ± 1.89)	15 (23.11 ± 2.97)	47	24 (23.57 ± 3.98)	14 (23.04 ± 2.25)	38
Total	102	81	183	76	65	141

### CBCT

All scans were taken by NewTom Scanner (NewTom AG, Marburg, Germany). Field of View was 15 × 15 cm, acquisition time was 3.6 s with a voxel size of 0.3 mm and exposure was set at 110 kVp and 2.03 mAs. CBCT images were saved as digital imaging and communications in medicine (DICOM) format and reconstructed in Dolphin 3D Imaging software (Version 11.8, Dolphin Imaging and Management Solutions, Chatsworth, Calif).

### Measurements

**Fig. 1:**
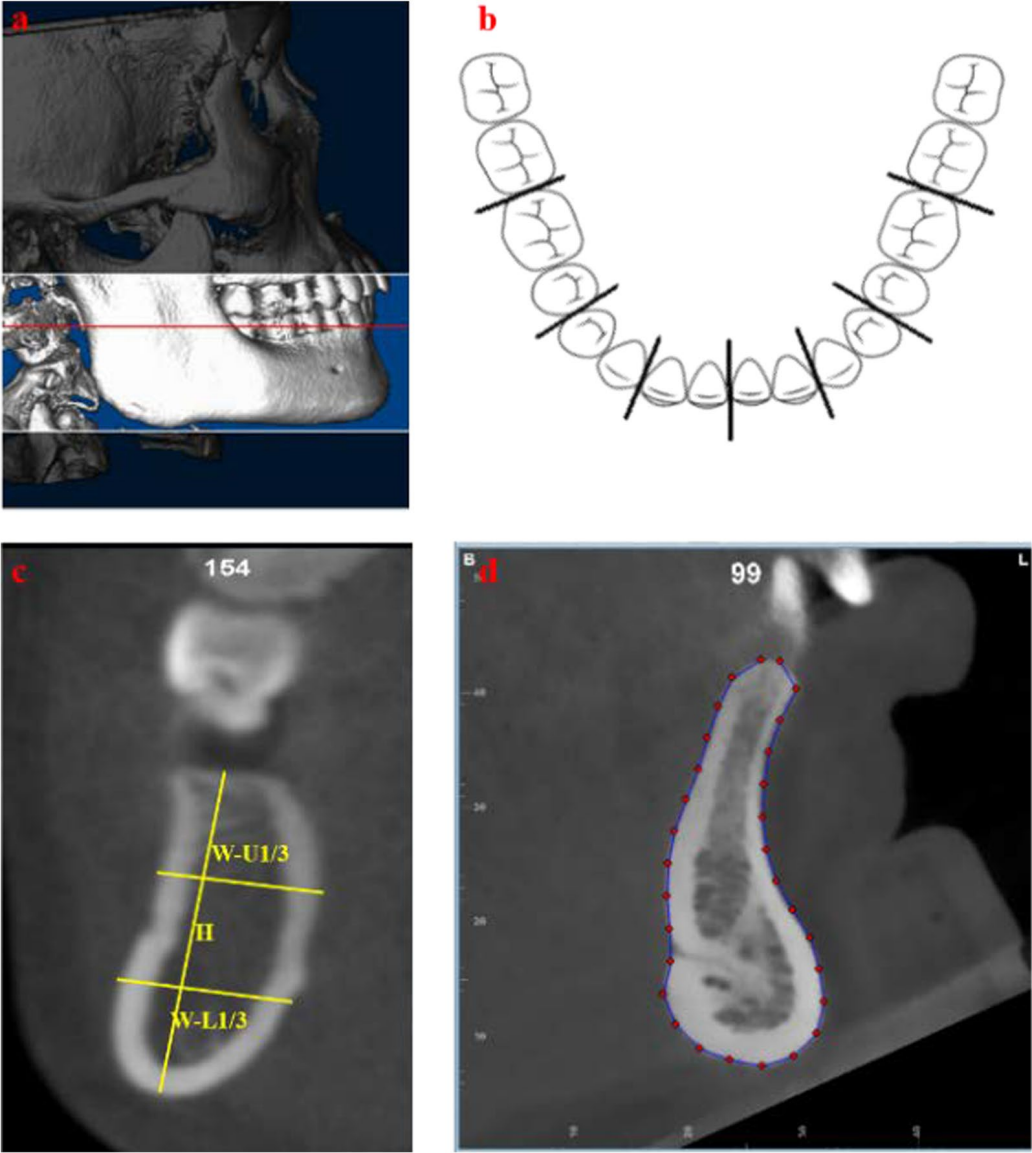
Measurements with Dolphin software and tpsDig2. **a** Overlapped inferior border of bilateral orbits and inferior and posterior border of mandible and reorientation of mandible in order to make mandibular plane parallel to horizontal. **b** Cross-section of mandible between central incisors, lateral incisor and canine, first and second premolars, first and second molars from left and right sides. **c** Measurements of mandibular cross-section. **d** Thirty landmarks along the outer cortex

Inferior border of bilateral orbits and bilateral inferior and posterior border of mandible were overlapped to make sure the proper head position to reestablish cephalograms [21, 22]. Mandible was reoriented in order that mandibular plane was parallel to the horizontal (Fig. 1a) [2, 23]. Then mandibular cross-sections between central incisors, lateral incisor and canine, first and second premolars, first and second molars from left and right sides were obtained and saved as JPEG files (Fig. 1b, c). Thirty points were set along the mandibular outer cortex equidistantly (Fig. 1d) using tps Utility program (Version 1.78, Rohlf, F.J., Department of Ecology and Evolution, State University of New York at Stony Brook) and tpsDig2 (Version 2.31). The buccal and lingual alveolar ridge points were both set as fixed points and 28 in-between landmarks were set as semi-landmarks using R studio (Version 1.3.1056) and they were able to slide along the curves until the TPS bending energy between the specimen and the Procrustes mean shape was minimal [12, 24]. GPA, CVA and DFA were conducted to depict the contour of mandibular cross-sections using MorphoJ (Version 1.07a, Faculty of Life Sciences, University of Manchester, Manchester, UK) and R studio. CVA was conducted at symphysis and molar region among different sagittal groups and DFA was conducted between Class I and Class II patients. Procrustes distance and Mahalanobis distance were calculated while *P* values were calculated from permutation tests and 10,000 and 1000 permutation rounds were carried out for CVA and DFA, respectively [25]. All measurements were listed in Table 3.

**Table 3:** Definitions of measurements

Measurement	Abbreviation	Definition
Mandibular height	H	The longest distance between midpoint of alveolar crest and inferior border of mandible
Upper third of mandibular width	W-U1/3	Distance between buccal/labial and lingual outer cortex in upper third of mandible, which is vertical to mandibular height mentioned above
Lower third of mandibular width	W-L1/3	Distance between buccal/labial and lingual outer cortex in lower third of mandible, which is vertical to mandibular height mentioned above

### Statistical analysis

Q–Q plot and Kolmogorov–Smirnov test in each group were carried out to ensure the variables met normal distribution and could be analyzed using parametric analysis. Paired-t test was used to compare the measurements between left and right sides and if no significant difference was found, an average value was used in further comparison. One-way analysis of variance (ANOVA) and Bonferroni post hoc test were used to compare the difference of measurements among different groups. ANOVA was also used to compared the difference of age among 12 groups. Statistical analysis was performed with IBM SPSS Statistics software (Version 20; IBM Corporation, NY, USA. SPSS, Inc., an IBM Company). Significance level was defined as *P* < 0.05.

Twenty CBCT images were chosen randomly and measured by two orthodontists (Chen HT and Hu XN) with 3 months interval to test inter- and intra-observer reliability. No significant difference of bilateral sides was found with paired-sample *t*-test, thus an average value of bilateral sides was used in the following comparisons.

## Results

No statistical difference of age was found among 12 groups. Gender difference was noted in majority of measurements in this study; therefore, females and males were analyzed separately in the following comparisons. Inter-examiner and intra-examiner coefficient correlation values were within acceptable range (Supplemental Table).

### Comparison of mandibular cross-sectional morphology from symphysis to molar region in different groups

Measurements of cross-sectional morphology for each group were shown in Table 4 and Table 5.

**Table 4:** Comparison of mandibular morphology in females

Index (Mean ± SD (mm))	Mandibular height					Upper third of mandibular width					Lower third of mandibular width				
	11H	23H	45H	67H	Comparison among regions	11 W-U1/3	23 W-U1/3	45 W-U1/3	67 W-U1/3	Comparison among regions	11 W-L1/3	23 W-L1/3	45 W-L1/3	67 W-L1/3	Comparison among regions
Class I normodivergent	31.30 ± 1.91	31.22 ± 1.74	30.09 ± 1.72	26.79 ± 1.68	11 = 23 = 45, 45 > 67***	7.86 ± 1.24	8.33 ± 1.31	10.66 ± 1.38	14.91 ± 1.66	11 = 23, 23 < 45***, 45 < 67***	13.35 ± 1.62	10.56 ± 1.27	10.60 ± 1.31	12.65 ± 1.16	11 > 23***, 23 = 45, 45 < 67***, 11 = 67
Class I hyperdivergent	33.24 ± 3.04	33.27 ± 2.86	32.14 ± 2.56	27.49 ± 2.12	11 = 23 = 45, 45 > 67***	6.92 ± 1.13	7.48 ± 1.23	10.21 ± 1.42	14.25 ± 1.37	11 = 23, 23 < 45***, 45 < 67***	12.69 ± 1.14	9.77 ± 1.01	10.59 ± 1.28	12.68 ± 1.40	11 > 23***, 23 = 45, 45 < 67***, 11 = 67
Class I hypodivergent	29.63 ± 2.58	29.64 ± 2.21	28.85 ± 2.30	27.58 ± 2.31	11 = 23 = 45 = 67	8.93 ± 1.55	9.40 ± 1.39	11.54 ± 1.61	15.44 ± 1.45	11 = 23, 23 < 45***, 45 < 67***	13.86 ± 1.49	11.02 ± 1.59	10.42 ± 1.48	12.93 ± 1.42	11 > 23***, 23 = 45, 45 < 67***, 11 = 67
Class II normodivergent	32.52 ± 2.71	31.78 ± 2.43	30.91 ± 2.39	28.23 ± 2.20	11 = 23 = 45, 45 > 67***	7.82 ± 1.28	8.58 ± 1.30	10.94 ± 1.33	15.11 ± 1.47	11 = 23, 23 < 45***, 45 < 67***	12.78 ± 1.65	10.64 ± 1.24	10.69 ± 1.25	12.82 ± 1.28	11 > 23***, 23 = 45, 45 < 67***, 11 = 67
Class II hyperdivergent	34.22 ± 2.64	33.38 ± 2.69	31.30 ± 2.45	26.84 ± 2.27	11 = 23, 23 > 45***, 45 > 67***	6.55 ± 1.34	7.06 ± 1.57	9.91 ± 1.48	14.19 ± 1.86	11 = 23, 23 < 45***, 45 < 67***	11.71 ± 1.65	9.51 ± 1.52	10.38 ± 1.34	12.03 ± 1.50	11 > 23***, 23 = 45, 45 < 67*, 11 = 67
Class II hypodivergent	30.16 ± 1.63	29.32 ± 1.47	28.37 ± 1.45	26.99 ± 2.01	11 = 23 = 45 = 67	9.50 ± 1.41	9.98 ± 1.49	12.06 ± 1.83	15.47 ± 1.85	11 = 23 = 45, 45 < 67***	14.29 ± 2.23	11.49 ± 1.94	10.77 ± 1.94	12.85 ± 1.71	11 > 23**, 23 = 45 = 67
Comparison among different vertical groups	1L < 1A**, 1L < 1H***, 1A < 1H**, 2L < 2A**, 2L < 2H***, 2A < 2H**	1L < 1A**, 1L < 1H***, 1A < 1H***, 2L < 2A**, 2L < 2H***, 2A < 2H**	1L < 1A*, 1L < 1H***, 1A < 1H***, 2L < 2A***, 2L < 2H***,	, 2H < 2A**		1L > 1A**, 1L > 1H***, 1A > 1H**, 2L > 2A***, 2L > 2H***, 2A > 2H***	1L > 1A**, 1L > 1H***, 1A > 1H*, 2L > 2A**, 2L > 2H***, 2A > 2H***	1L > 1A*, 1L > 1H***, 2L > 2A*, 2L > 2H***, 2A > 2H**	1L > 1H**, 2L > 2H*, 2A > 2H*		1L > 1H**, 2L > 2A**, 2L > 2H***, 2A > 2H**	1L > 1H***, 1A > 1H*, 2L > 2H**, 2A > 2H**	NS	2A > 2H*	
Comparison between different sagittal groups	1A < 2A*, 1L = 2L 1H = 2H	1A = 2A 1L = 2L 1H = 2H	1A = 2A, 1L = 2L, 1H = 2H	1A < 2A**, 1L = 2L, 1H = 2H		1A = 2A, 1L = 2L, 1H = 2H	1A = 2A, 1L = 2L, 1H = 2H	1A = 2A, 1L = 2L, 1H = 2H	1A = 2A, 1L = 2L, 1H = 2H		1A = 2A, 1L = 2L, 1H > 2H*	1A = 2A, 1L = 2L, 1H = 2H	1A = 2A, 1L = 2L, 1H = 2H	1A = 2A, 1L = 2L, 1H = 2H	

1A: Class I normodivergent group; 1L: Class I hypodivergent group; 1H: Class I hyperdivergent group;

2A: Class II normodivergent group; 2L: Class II hypodivergent group; 2H: Class II hyperdivergent group

^*^P < 0.05, **P < 0.01, ***P < 0.001; NS: Not significant

**Table 5:** Comparison of mandibular morphology in males

Index (Mean ± SD (mm))	Mandibular height					Upper third of mandibular width					Lower third of mandibular width				
	11H	23H	45H	67H	Comparison among regions	11 W-U1/3	23 W-U1/3	45 W-U1/3	67 W-U1/3	Comparison among regions	11 W-L1/3	23 W-L1/3	45 W-L1/3	67 W-L1/3	Comparison among regions
Class I normodivergent	35.19 ± 3.10	34.55 ± 3.10	33.57 ± 2.79	29.85 ± 2.41	11 = 23 = 45, 45 > 67***	8.33 ± 1.49	9.25 ± 1.66	12.13 ± 1.67	15.53 ± 2.00	11 = 23, 23 < 45***, 45 < 67***	14.66 ± 1.92	11.46 ± 1.75	11.55 ± 1.68	13.47 ± 1.92	11 > 23***, 23 = 45, 45 < 67*, 11 = 67
Class I hyperdivergent	36.58 ± 3.25	36.11 ± 3.18	34.84 ± 2.98	29.37 ± 2.60	11 = 23 = 45, 45 > 67***	7.65 ± 1.11	8.48 ± 1.11	11.54 ± 1.52	14.72 ± 1.46	11 = 23, 23 < 45***, 45 < 67***	13.86 ± 1.42	10.54 ± 1.23	10.88 ± 1.41	13.01 ± 1.29	11 > 23***, 23 = 45, 45 < 67*, 11 = 67
Class I hypodivergent	33.28 ± 2.86	32.81 ± 2.44	31.55 ± 2.09	29.62 ± 2.15	11 = 23 = 45, 45 > 67*	8.93 ± 1.33	9.62 ± 1.31	12.32 ± 1.72	15.35 ± 1.95	11 = 23, 23 < 45***, 45 < 67***	14.55 ± 2.11	11.90 ± 1.97	11.08 ± 1.92	12.93 ± 1.57	11 > 23***, 23 = 45 = 67
Class II normodivergent	35.44 ± 2.63	34.89 ± 2.08	33.45 ± 2.04	30.34 ± 2.26	11 = 23 = 45, 45 > 67***	8.41 ± 1.35	9.28 ± 1.53	12.02 ± 1.57	15.36 ± 1.90	11 = 23, 23 < 45***, 45 < 67***	13.61 ± 1.82	11.07 ± 1.65	10.94 ± 1.65	12.54 ± 1.82	11 > 23***, 23 = 45 = 67
Class II hyperdivergent	37.59 ± 2.50	36.39 ± 2.53	34.59 ± 2.19	29.98 ± 1.54	11 = 23 = 45, 45 > 67***	7.55 ± 1.16	8.54 ± 1.61	11.49 ± 1.61	14.86 ± 1.79	11 = 23, 23 < 45***, 45 < 67***	12.57 ± 1.75	10.09 ± 1.94	10.12 ± 2.27	11.98 ± 1.69	11 > 23***, 23 = 45, 45 < 67*, 11 = 67
Class II hypodivergent	34.74 ± 2.52	32.95 ± 3.28	31.88 ± 2.56	30.83 ± 1.57	11 = 23 = 45 = 67	9.51 ± 1.53	9.95 ± 1.50	12.08 ± 1.77	15.92 ± 1.75	11 = 23 = 45, 45 < 67***	14.25 ± 1.64	11.31 ± 1.17	10.23 ± 1.55	13.11 ± 1.35	11 > 23**, 23 = 45, 45 < 67**, 11 = 67
Comparison among different vertical groups	1L < 1A*, 1L < 1H***, 2L < 2H**, 2A < 2H**	1L < 1A*, 1L < 1H***, 1A < 1H*, 2L < 2A*, 2L < 2H***	1L < 1A**, 1L < 1H***, 2L < 2H**	NS		1L > 1H**, 2L > 2A*, 2L > 2H***, 2A > 2H*	1L > 1H*, 2L > 2H**	NS	NS		2L > 2H**, 2A > 2H*,	1L > 1H**, 2L > 2H*, 2A > 2H*	NS	2L > 2H*	
Comparison between different sagittal groups	1A = 2A 1L = 2L 1H = 2H	1A = 2A 1L = 2L 1H = 2H	1A = 2A 1L = 2L 1H = 2H	1A = 2A 1L = 2L 1H = 2H		1A = 2A 1L = 2L 1H = 2H	1A = 2A 1L = 2L 1H = 2H	1A = 2A 1L = 2L 1H = 2H	1A = 2A 1L = 2L 1H = 2H		1A > 2A* 1L = 2L 1H > 2H*	1A = 2A 1L = 2L 1H = 2H	1A = 2A 1L = 2L 1H = 2H	1A > 2A*, 1L = 2L 1H > 2H*	

1A: Class I normodivergent group; 1L: Class I hypodivergent group; 1H: Class I hyperdivergent group;

2A: Class II normodivergent group; 2L: Class II hypodivergent group; 2H: Class II hyperdivergent group

^*^P < 0.05, **P < 0.01, ***P < 0.001; NS: Not significant

Table 4 Comparison of mandibular morphology in females.

Table 5 Comparison of mandibular morphology in males.

#### Mandibular height

In Class I and Class II hyperdivergent and normodivergent groups, mandibular height decreased from symphysis to molar region, and significant lowest height was noted in the molar region (*P* < 0.05). But no significant difference was found for mandibular height from symphysis to molar region in all subjects in Class II hypodivergent group and females in Class I hypodivergent group.

#### Upper third of mandibular width

Upper third of mandibular width increased significantly from the canine region to the molar region with the widest width in the molar region (*P* < 0.05). But no significant difference was noted from symphysis to the premolar region in Class II hypodivergent group.

#### Lower third of mandibular width

Generally, lower third of mandibular width decreased from symphysis to the canine region, and increased from the premolar region to the molar region in all groups, while the average value of lower third of mandibular width at symphysis was similar to that in the molar region, so as to the width in the canine region compared with that in the premolar region (*P* > 0.05).

However, no significant difference width from the canine to the molar region was noted in males in Class I hypodivergent and Class II normodivergent groups and females in Class II hypodivergent group.

#### Ratio between height and width

Generally, the ratio between mandibular height and upper third width decreased from symphysis to the molar region in all groups. However, the ratio between mandibular height and lower third width was the biggest in the canine region in Class I or Class II normodivergent and hyperdivergent groups. But in Class I or Class II hypodivergent group, the ratio between height and lower third width was the biggest in the premolar region (Table 6).

**Table 6:** Comparison of ratio between mandibular height and width in different groups

Gender	Ratio	Class I normo-divergent	Class I hyper-divergent	Class I hypo-divergent	Class II normo-divergent	Class II hyper-divergent	Class II hypo-divergent	Comparison
Male	11H/11 W-U1/3	4.37 ± 0.93	4.91 ± 1.01	3.80 ± 0.60	4.32 ± 0.73	5.11 ± 0.96	3.80 ± 0.98	2L < 2H***, 1L < 1H**
	11H/11 W-L1/3	2.43 ± 0.31	2.67 ± 0.37	2.33 ± 0.37	2.63 ± 0.27	3.06 ± 0.55	2.49 ± 0.51	2L < 2H**, 1H < 2H**, 2A < 2H**
	23H/23 W-U1/3	3.86 ± 0.79	4.35 ± 0.82	3.46 ± 0.45	3.86 ± 0.69	4.42 ± 0.95	3.44 ± 0.90	2L < 2H*, 1L < 1H**
	23H/23 W-L1/3	3.08 ± 0.50	3.48 ± 0.59	2.82 ± 0.44	3.21 ± 0.47	3.75 ± 0.84	2.97 ± 0.62	2L < 2H**, 1L < 1H**, 2A < 2H*
	45H/45 W-U1/3	2.82 ± 0.46	3.06 ± 0.42	2.60 ± 0.35	2.83 ± 0.43	3.07 ± 0.51	2.71 ± 0.53	1L < 1H*
	45H/45 W-L1/3	2.96 ± 0.44	3.27 ± 0.62	2.92 ± 0.46	3.12 ± 0.47	3.62 ± 1.00	3.20 ± 0.63	2A < 2H*
	67H/67 W-U1/3	1.95 ± 0.28	2.01 ± 0.27	1.95 ± 0.23	2.00 ± 0.25	2.04 ± 0.26	1.96 ± 0.27	NS
	67H/67 W-L1/3	2.25 ± 0.33	2.28 ± 0.34	2.32 ± 0.28	2.46 ± 0.35	2.55 ± 0.38	2.38 ± 0.29	NS
Female	11H/11 W-U1/3	4.08 ± 0.71	4.94 ± 1.01	3.43 ± 0.74	4.29 ± 0.89	5.42 ± 1.11	3.24 ± 0.47	2L < 2A*, 2L < 2H***, 1L < 1A**, 1L < 1H***, 1A < 1H**, 2A < 2H***
	11H/11 W-L1/3	2.38 ± 0.33	2.64 ± 0.35	2.16 ± 0.32	2.59 ± 0.43	2.97 ± 0.43	2.15 ± 0.29	2L < 2A*, 2L < 2H***,1L < 1H***, 1H < 2H*, 2A < 2H**
	23H/23 W-U1/3	3.83 ± 0.61	4.57 ± 0.93	3.32 ± 0.59	3.79 ± 0.66	4.93 ± 1.07	2.99 ± 4.14	2L < 2H***, 1L < 1H***, 1A < 1H**, 2A < 2H***
	23H/23 W-L1/3	3.00 ± 0.41	3.43 ± 0.40	2.75 ± 0.47	3.02 ± 0.38	3.60 ± 0.62	2.61 ± 0.40	2L < 2H***, 1L < 1H***, 1A < 1H*, 2A < 2H**
	45H/45 W-U1/3	2.86 ± 0.37	3.20 ± 0.43	2.54 ± 0.36	2.86 ± 0.93	3.23 ± 0.56	2.39 ± 0.32	2L < 2A*, 2L < 2H***, 1L < 1H***, 2A < 2H*
	45H/45 W-L1/3	2.88 ± 0.39	3.08 ± 0.45	2.82 ± 0.46	2.93 ± 0.41	3.06 ± 0.42	2.70 ± 0.43	NS
	67H/67 W-U1/3	1.82 ± 0.21	1.95 ± 0.23	1.80 ± 0.20	1.88 ± 0.19	1.92 ± 0.36	1.76 ± 0.16	NS
	67H/67 W-L1/3	2.13 ± 0.23	2.19 ± 0.28	2.15 ± 0.27	2.21 ± 0.20	2.26 ± 0.28	2.12 ± 0.20	NS

1A: Class I normodivergent group; 1L: Class I hypodivergent group; 1H: Class I hyperdivergent group;

2A: Class II normodivergent group; 2L: Class II hypodivergent group; 2H: Class II hyperdivergent group

^*^P < 0.05; **P < 0.01; ***P < 0.001; NS: Not significant

In males and females, the ratio between height and upper/lower third width was the biggest in hyperdivergent groups in all regions (*P* < 0.05, Table 6). Additionally, for males and females, the ratio between height and lower third width at symphysis was significantly higher in Class II hyperdivergent group than that in Class I hyperdivergent group (*P* < 0.05, Table 6).

Table 6 Comparison of ratio between mandibular height and width in different groups.

#### Ratio between upper 1/3 and lower 1/3 of mandibular width

Both in males and females, the ratio between upper 1/3 width and lower 1/3 width increased from the symphysis to the molar region in all different groups. The average value of the ratio in Class I hyperdivergent group was significantly lower than that in Class II hyperdivergent group (*P* < 0.05, Table 7).

**Table 7:** Comparison of ratio between upper 1/3 and lower 1/3 of mandibular width

Gender	Ratio	Class I normodivergent	Class I hyperdivergent	Class I hypodivergent	Class II normodivergent	Class II hyperdivergent	Class II hypodivergent	Comparison
Male	11	0.57 ± 0.08	0.55 ± 0.07	0.62 ± 0.07	0.62 ± 0.09	0.60 ± 0.08	0.67 ± 0.06	2L > 2H**
	23	0.81 ± 0.09	0.81 ± 0.11	0.82 ± 0.09	0.85 ± 0.13	0.86 ± 0.14	0.88 ± 0.06	NS
	45	1.06 ± 0.14	1.08 ± 0.22	1.12 ± 0.12	1.11 ± 0.13	1.19 ± 0.31	1.19 ± 0.11	NS
	67	1.16 ± 0.10	1.13 ± 0.09	1.19 ± 0.07	1.23 ± 0.11	1.25 ± 0.09	1.21 ± 0.03	2H > 1H**
	Comparison	11 < 23 < 45*** 45 < 67**	11 < 23 < 45*** 45 = 67	11 < 23 < 45*** 45 = 67	11 < 23 < 45*** 45 < 67**	11 < 23 < 45*** 45 = 67	11 < 23 < 45*** 45 = 67	
Female	11	0.59 ± 0.06	0.55 ± 0.07	0.64 ± 0.08	0.61 ± 0.07	0.56 ± 0.08	0.67 ± 0.08	2L > 2H***, 1L > 1H***
	23	0.79 ± 0.08	0.76 ± 0.09	0.86 ± 0.09	0.81 ± 0.08	0.74 ± 0.10	0.88 ± 0.09	2L > 2H**, 1L > 1H*
	45	1.01 ± 0.12	0.97 ± 0.15	1.12 ± 0.14	1.03 ± 0.16	0.96 ± 0.11	1.13 ± 0.08	2L > 2H*, 1L > 1H*
	67	1.18 ± 0.10	1.13 ± 0.10	1.20 ± 0.12	1.18 ± 0.10	1.18 ± 0.09	1.21 ± 0.09	NS
	Comparison	11 < 23 < 45 < 67***	11 < 23 < 45 < 67***	11 < 23 < 45*** 45 < 67*	11 < 23 < 45 < 67***	11 < 23 < 45 < 67***	11 < 23 < 45*** 45 = 67	

1A: Class I normodivergent group; 1L: Class I hypodivergent group; 1H: Class I hyperdivergent group;

2A: Class II normodivergent group; 2L: Class II hypodivergent group; 2H: Class II hyperdivergent group

^*^P < 0.05, **P < 0.01, ***P < 0.001; NS: Not significant

Table 7 Comparison of ratio between upper 1/3 and lower 1/3 of mandibular width.

### Sagittal influence on mandibular cross-sectional morphology

#### Mandibular height

No significant difference was noted in mandibular height from symphysis to the premolar region when compared Class I with Class II groups with similar vertical dimensions, except for normodivergent females in symphysis and molar region, with the higher average value in Class II group (*P* < 0.05).

#### Upper third of mandibular width

When compared Class I with Class II groups, no significant difference was noted in upper third width in hyperdivergent, normodivergent or hypodivergent group.

#### Lower third of mandibular width

In females, no significant difference was noted in lower third of mandibular width when compared between Class I and Class II groups with similar vertical dimensions, except for that in the symphysis, with significantly wider lower third width in Class I hyperdivergent group than that in Class II hyperdivergent group (*P* < 0.05).

However, in males, Class I normodivergent and hyperdivergent groups showed significantly wider lower third width in symphysis and the molar region than that in Class II normodivergent and hyperdivergent groups (*P* < 0.05).

### Vertical influence on mandibular cross-sectional morphology

#### Mandibular height

From symphysis to the premolar region, mandibular height in hyperdivergent groups was significantly higher than that in normodivergent and hypodivergent groups (*P* < 0.05), except for the molar region.

#### Upper third of mandibular width

Subjects in hypodivergent groups showed significantly wider upper third of mandibular width from symphysis to the molar region than that in hyperdivergent group (*P* < 0.05), except for the premolar and molar regions when compared different male groups (*P* > 0.05).

#### Lower third of mandibular width

Generally, males and females in hypodivergent groups showed wider lower third width than that in normodivergent and hyperdivergent groups at symphysis and the canine region. Additionally, males and females in Class II hyperdivergent group showed narrowest lower third width in the molar region when compared with other groups.

### Visualized mandibular morphology by GPA, CVA and DFA

#### Geometric morphometric measurement by GPA from symphysis to molar region

**Fig. 2:**
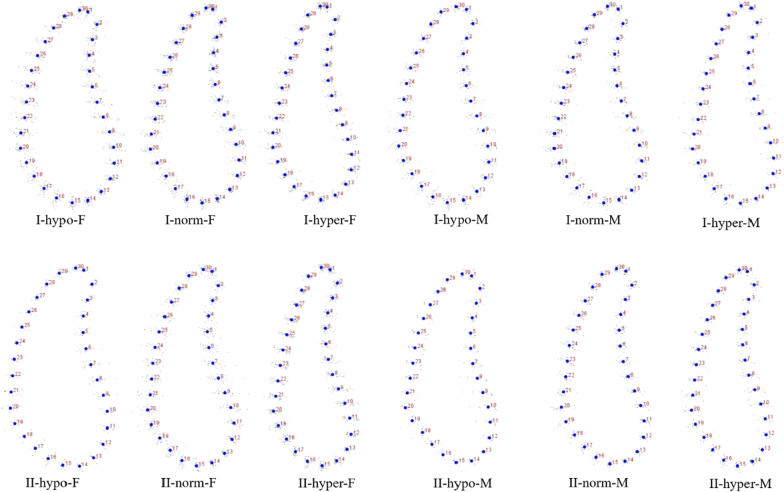
GPA of symphysis. I: Class I; II: Class II; M: male; F: female; hypo: hypodivergent; hyper: hyperdivergent; norm: normodivergent

**Fig. 3:**
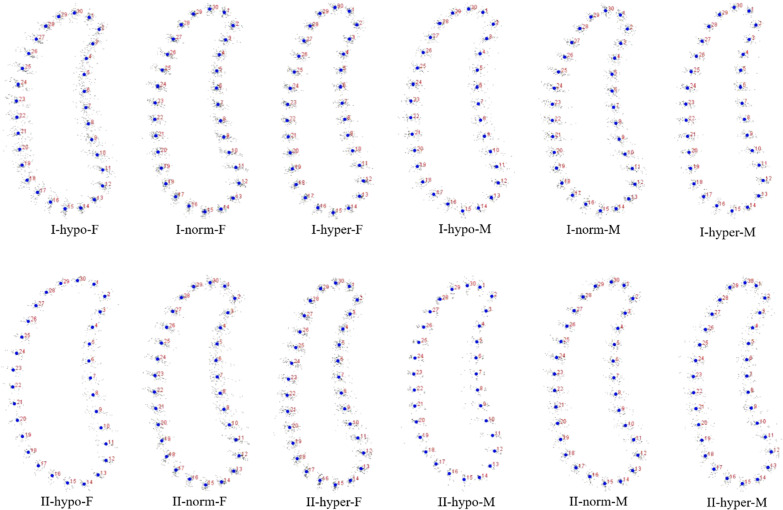
GPA of canine region. I: Class I; II: Class II; M: male; F: female; hypo: hypodivergent; hyper: hyperdivergent; norm: normodivergent

**Fig. 4:**
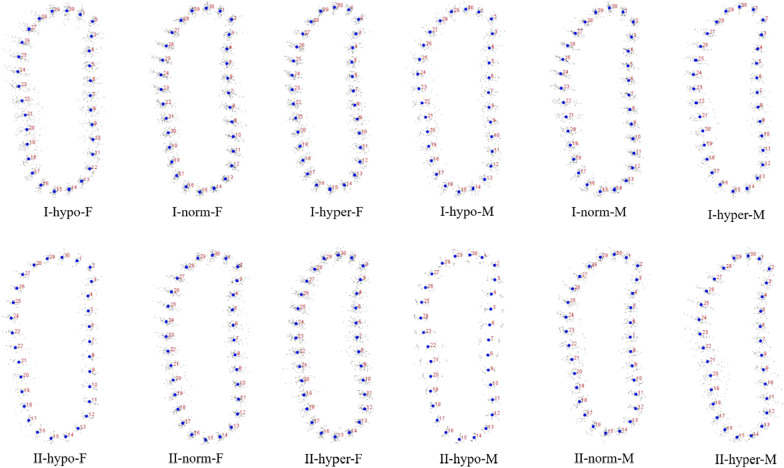
GPA of premolar region. I: Class I; II: Class II; M: male; F: female; hypo: hypodivergent; hyper: hyperdivergent; norm: normodivergent

**Fig. 5:**
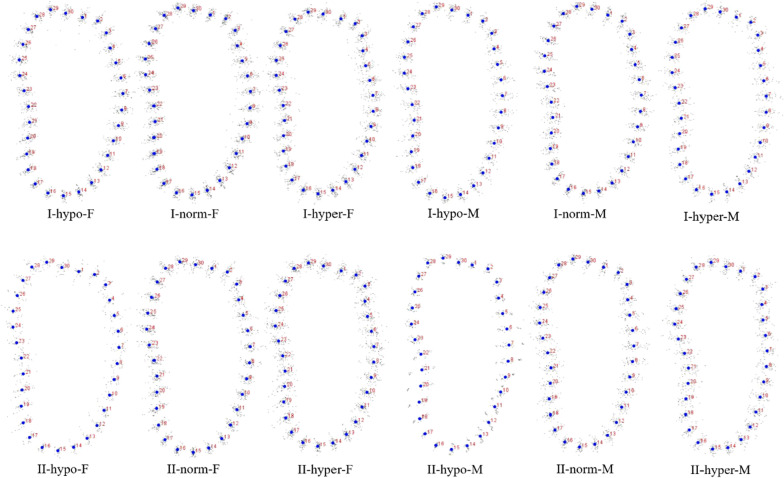
GPA of molar region. I: Class I; II: Class II; M: male; F: female; hypo: hypodivergent; hyper: hyperdivergent; norm: normodivergent

The coordinates from all the patients in each group were calculated to get the mean shape using GPA. The result of GPA of each group was shown in Figs. 2, 3, 4 and 5. Procrustes distance of each group was shown in Table 8.

**Table 8:** Procrustes distance in each group

Region	Gender	Group	Class I hypodivergent	Class II hypodivergent	Class I normodivergent	Class I hyperdivergent	Class II normodivergent
Symphysis	Male	Class II hypodivergent	0.0382				
		Class I normodivergent	0.0336*				
		Class I hyperdivergent	0.0700***		0.0411**		
		Class II normodivergent		0.0369*	0.0346**		
		Class II hyperdivergent		0.0771***		0.0363*	0.0503***
	Female	Class II hypodivergent	0.0282				
		Class I normodivergent	0.0490***				
		Class I hyperdivergent	0.0942***		0.0470***		
		Class II normodivergent		0.0623**	0.0253		
		Class II hyperdivergent		0.1324***		0.0312**	0.0744***
Canine region	Male	Class II hypodivergent	0.0246				
		Class I normodivergent	0.0264*				
		Class I hyperdivergent	0.0575***		0.0397***		
		Class II normodivergent		0.0361**	0.0241*		
		Class II hyperdivergent		0.0575***		0.0299**	0.0395***
	Female	Class II hypodivergent	0.0263				
		Class I normodivergent	0.0515***				
		Class I hyperdivergent	0.0924***		0.0423***		
		Class II normodivergent		0.0547***	0.0135		
		Class II hyperdivergent		0.1177***		0.0222*	0.0650***
Premolar region	Male	Class II hypodivergent	0.0324*				
		Class I normodivergent	0.0285**				
		Class I hyperdivergent	0.0435***		0.0301**		
		Class II normodivergent		0.0317*	0.0219		
		Class II hyperdivergent		0.0452**		0.0265	0.0323**
	Female	Class II hypodivergent	0.0287				
		Class I normodivergent	0.0414***				
		Class I hyperdivergent	0.0701***		0.0307***		
		Class II normodivergent		0.0519***	0.0124		
		Class II hyperdivergent		0.0932***		0.0113	0.0429***
Molar region	Male	Class II hypodivergent	0.0192				
		Class I normodivergent	0.0117				
		Class I hyperdivergent	0.0151		0.0131		
		Class II normodivergent		0.0248	0.0268*		
		Class II hyperdivergent		0.0420**		0.0366**	0.0253*
	Female	Class II hypodivergent	0.0189				
		Class I normodivergent	0.0124				
		Class I hyperdivergent	0.0298***		0.0262***		
		Class II normodivergent		0.0292*	0.0184*		
		Class II hyperdivergent		0.0352**		0.0125	0.0160

^*^P < 0.05, **P < 0.01, ***P < 0.001

Table 8 Procrustes distance in each group.

#### CVA and DFA of symphysis and molar region

**Fig. 6:**
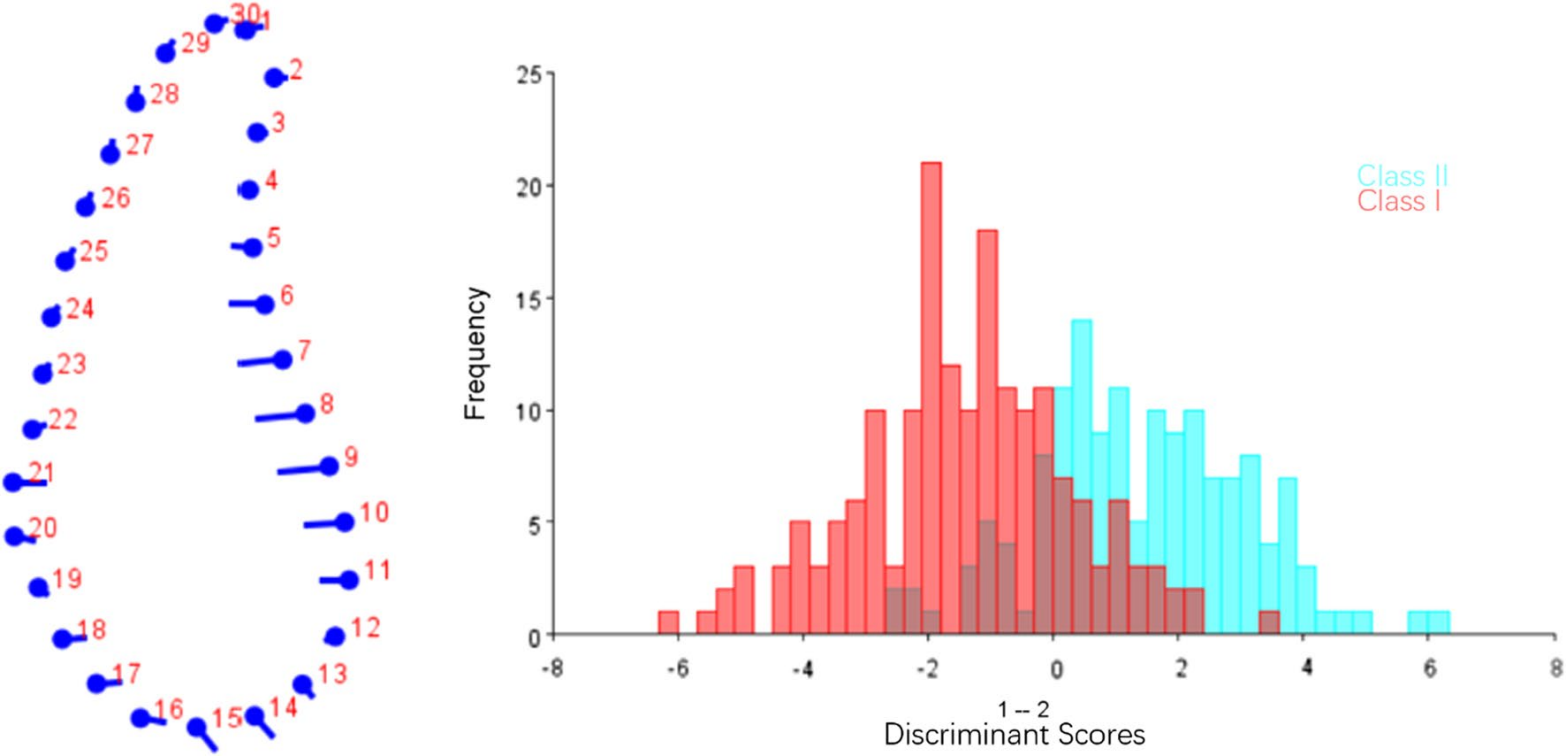
DFA of symphysis between Class I and Class II. The dots on the left figure represent the negative axis on the right figure, and the ends of the line represent the positive axis

**Fig. 7:**
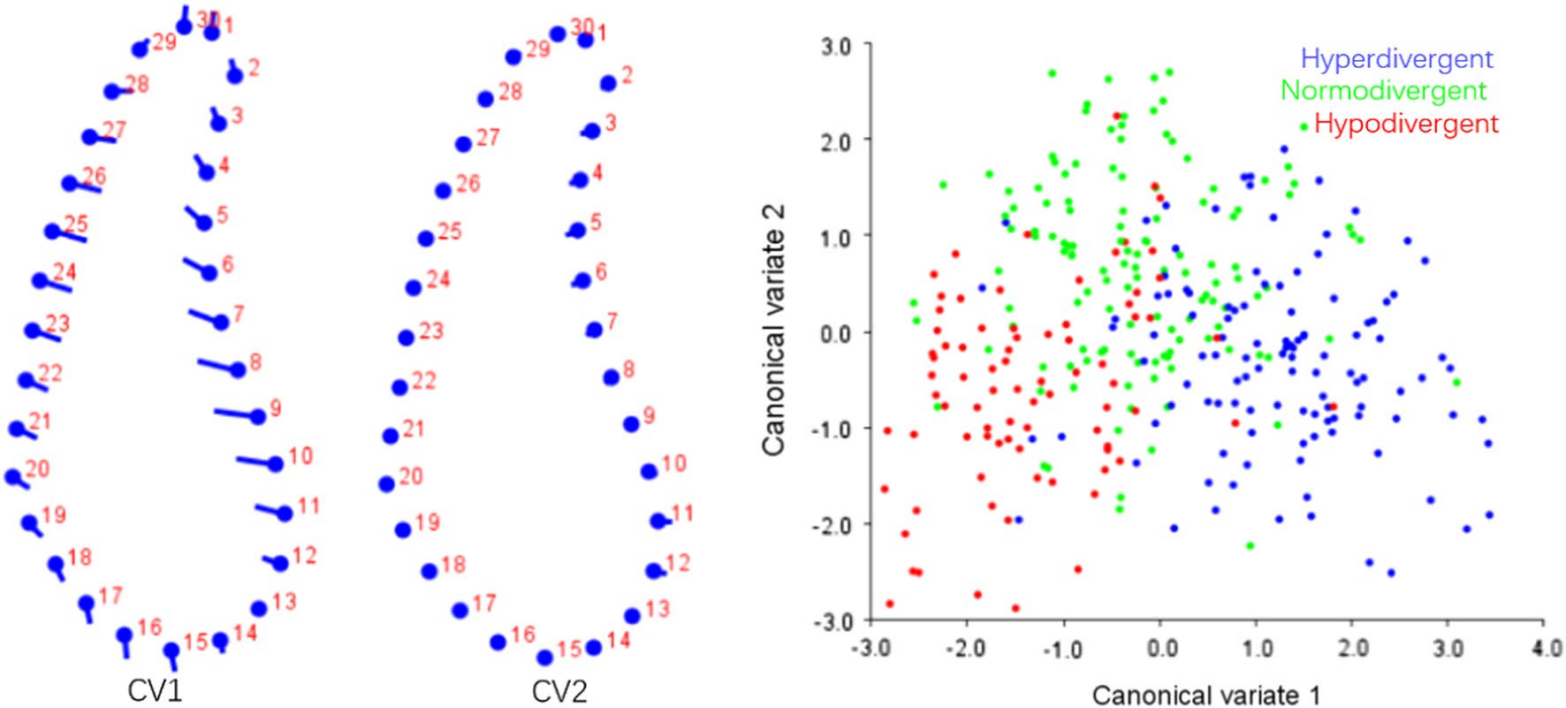
CVA of symphysis among different vertical dimensions. Variance of CV1 and CV2 accounted for 77.44% and 22.56% of total variance, respectively

**Fig. 8:**
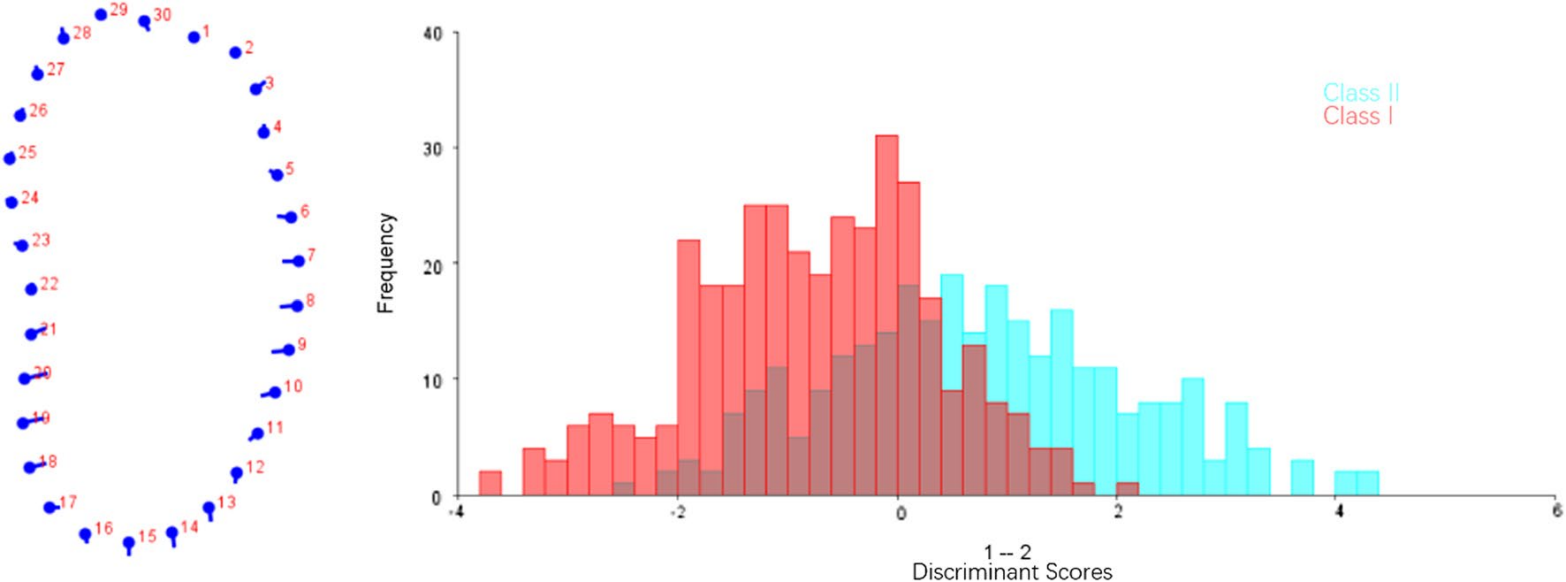
DFA of molar region between Class I and Class II. The dots on the left figure represent the negative axis on the right figure, and the ends of the line represent the positive axis

**Fig. 9:**
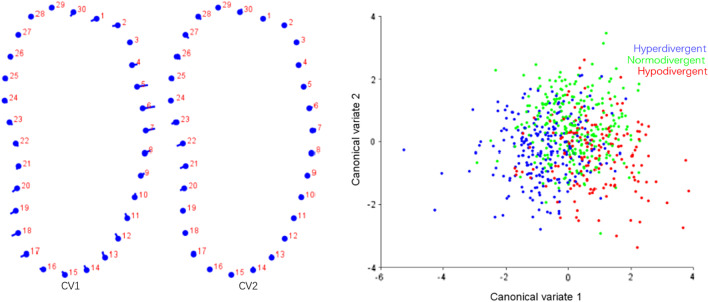
CVA of molar region among different vertical dimensions. Variance of CV1 and CV2 accounted for 72.98% and 27.02% of total variance, respectively

The results of DFA of symphysis and molar region in Class I and Class II were shown in Figs. 6 and 8. The results of CVA of symphysis and molar region among different vertical dimensions were listed in Figs. 7 and 9. Procrustes and Mahalanobis distance and the *P* value were listed in Table 9. CV1 is the direction with the most obvious morphological difference, while CV2 is the direction with the second most obvious morphological difference after removal of CV1. The dot in CV means the negative axis, and the end of the bar in CV means the positive axis.

**Table 9:** Procrustes and Mahalonobis distance in CVA in symphysis and molar region

	Class I vs Class II	Hyperdivergent vs normodivergent	Normodivergent vs hypodivergent	Hyperdivergent vs hypodivergent
**Symphysis**				
Procrustes distance	0.03***	0.05***	0.04***	0.09***
Mahalanobis distance	1.68***	1.75***	1.66***	2.57***
**Molar region**				
Procrustes distance	0.02***	0.02***	0.01	0.03***
Mahalanobis distance	1.23***	1.15***	1.15***	1.65***

***P < 0.001

In symphysis, main difference between Class I and Class II patients was noted along the anterior contour of symphysis. Class II subjects showed deeper curve along the anterior contour of symphysis (Fig. 6). CV1 in CVA of symphysis among different vertical patterns accounted for 77.44% of total variance, which showed hyperdivergent group had a narrower and higher symphysis and hypodivergent group had a wider and shorter symphysis, while little difference was found in CV2 (Fig. 7). The results of geometric morphometric measurement were in consistent with linear measurement mentioned above.

In molar region, Class II group showed a slightly narrower cross-section in the lower third than Class I group (Fig. 8).

## Discussion

### The influence of vertical facial pattern on mandibular cross-sectional morphology using linear measurement

Our results agreed with the previous studies that vertical facial pattern was the major factor to influence mandibular cross-sectional morphology, with significantly increased mandibular height in hyperdivergent groups [4, 23]. Growth pattern and aging factor might contribute to such difference, and increased mandibular height in anterior region also compensated for mandibular clockwise rotation in hyperdivergent subjects [23]. Based on present study, upper third of mandibular width in symphysis and canine region was significantly narrower in Class I and Class II hyperdivergent groups, which was consistent with the previous studies, Swasty et al. [4], Sadek et al. [5] and Klinge et al. [23] found hyperdivergent group had significantly narrower mandibular cross-section compared to normodivergent and hypodivergent groups, especially in anterior region. Sadek et al. [5] found hyperdivergent subjects showed narrower mandible in almost all sites. Thus, the clinical interpretation of these findings indicates that tipping movement of mandibular incisors in skeletal Class II hyperdivergent subjects to compromise the retrusive mandible needs to be carried out carefully. Configuration of symphysis should be considered when making treatment plan for hyperdivergent subjects.

### The influence of sagittal facial pattern on mandibular cross-sectional morphology using linear measurement

Sagittal facial pattern was found to have little influence on the mandibular cross-sectional morphology, especially when linear measurements were conducted, which was in accordance with the former studies [4, 5, 23]. However, it was interesting to find that when the measurement of ratio between height and width was performed and compared among the different groups, sagittal skeletal pattern did influence the mandibular cross-sectional shape. It was suggested that the subjects in Class II hyperdivergent group showed significantly increased ratio of mandibular height to lower third width than that in Class I hyperdivergent group (*P* < 0.05, Table 6), indicating a much “slimer” symphysis in Class II hyperdivergent subjects. The ratio between upper third and lower third mandibular width increased form symphysis the molar region in different groups, indicating an eventually narrower tendency towards the bottom of mandibular cross-section from anterior to posterior segment.

### The influence of vertical and sagittal facial pattern on mandibular cross-sectional morphology using statistical shape analysis

Statistical shape analysis has been used on the mandibular morphology study for several years. Apart from Ahn et al. [10, 11], Gomez et al. [26] used PCA to investigate the relationship between facial pattern and symphysis morphology and found the symphysis morphology was associated with vertical dimension and genders. They also demonstrated that combination of sagittal and vertical dimension was more significant using PCA compared to sagittal or vertical dimension alone with Pearson`s correlation test. In the present study, GPA gave us a more visualized mandibular cross-sectional morphology in different groups (Figs. 2, 3, 4, 5), which was in consistency with the results of the comparisons with linear measurement. However, more detailed characteristics could be illustrated in figures and provided a supplemental information to the results obtained from traditional linear measurements. The most remarkable features for symphysis were a deep curve along the anterior contour of symphysis in Class II compared with that in Class I group, and more convex along the posterior contour of symphysis in hypodivergent groups (Figs. 2, 6, 7). These characteristics, when together with the buccally inclined lower incisor during the compromised orthodontic treatment for the subjects with skeletal Class II and retrusive mandible, will result in a deeper mentolabial sulcus. In the molar region, flatter surface was noted along the anterior contour of mandibular cross-section in hyperdivergent groups (Figs. 5, 9).

### The clinical implications and prospect of the study

Mandibular morphology could be influenced by genetic factors, function of stomatognathic system, skeletal patterns and attachment of masticatory muscle to mandible [4, 27, 28]. Understanding of mandibular cross-sectional morphology could assist in making treatment plan, reduce the probability of iatrogenic damage to root and periodontal tissue during tooth movement in orthodontic treatment [4, 5, 8, 11, 29].

It has been proved that leveling in lower arch in hypodivergent patients occurs through buccal movement of anterior teeth [30]. Such buccal inclination could also occur in the aligning of anterior teeth without premolar extraction or dental compensation for Class II patients, which could present high risks of dehiscence and fenestration in anterior segments of mandible [31]. Prediction of the probability of periodontal damage at mandibular incisor region plays an important role in making treatment plan. Cook et al. [32] found the periodontal biotype was significantly related to labial plate thickness. Therefore, assessment of mandibular morphology is essential to avoid iatrogenic damage.

The present study focused on the comparisons of cross-sectional mandibular morphology between Class I and Class II young adults. The influence of growth and aging on mandibular morphology is still unclear and should be investigated in the further study. Besides, cross-sectional mandibular morphology of skeletal Class III patients also needs to be explored, and a longitudinal study before and after orthodontic treatment is also needed, which could explore the influence of tooth moving on the alveolar bone thickness, including buccal inclination of mandibular anterior teeth while aligning or leveling the lower arch, lingual inclination of mandibular anterior teeth while decompensation in skeletal Class II patients with mandible retrusion before orthognathic surgery, or retraction of anterior teeth after extraction of four premolars.

## Conclusions

1. The influence of vertical facial patterns on mandibular cross-sectional morphology is more obvious than that of sagittal skeletal pattern.
2. Subjects with increased vertical dimension presented with a remarkable “slimer” mandibular cross-sectional morphology at symphysis
3. A deeper curve along the anterior contour of symphysis in Class II hyperdivergent group was noted with GPA.

## Supplementary Information

**Additional file 1**. Inter and intra-observer correlation coefficient.

## Data Availability

The dataset used and/or analyzed during the current study are available from the corresponding authors on reasonable request.

## Abbreviations

CBCT: Cone-beam computed tomography
ANOVA: One-way analysis of variance
GPA: General Procrustes analysis
PCA: Principle component analysis
CVA: Canonical variance analysis
DFA: Discriminant function analysis
ANB: Angle formed by A-point–Nasion–B-point
SNB: Angle formed by Sella–Nasion–B-point
DICOM: Digital imaging and communications in medicine
MP: Mandibular plane
SPSS: Statistical package for social science
