# Supplementary material for: Comparison of mandibular cross-sectional morphology between Class I and Class II subjects with different vertical patterns: based on CBCT images and statistical shape analysis
Source: BMC Oral Health. 2021 May 5;21:238. doi: 10.1186/s12903-021-01591-3 (PMC8097981; doi:10.1186/s12903-021-01591-3)
Supplement: Supplementary file 1 — Additional file 1. Inter and intra-observer correlation coefficient. [file 12903_2021_1591_MOESM1_ESM.docx]

Supplemental Table A. Inter-observer correlation coefficient.

|  | **SNA (°)** | **SNB (°)** | **ANB (°)** | **Wits (mm)** | **MPSN (°)** | **FMA (°)** |  |
| --- | --- | --- | --- | --- | --- | --- | --- |
| ICC | 0.905 | 0.981 | 0.962 | 0.974 | 0.969 | 0.927 |  |
| 95%CI | 0.778~0.961 | 0.948~0.993 | 0.908~0.985 | 0.931~0.990 | 0.697~0.992 | 0.825~0.970 |  |
| p | 0.000 | 0.000 | 0.000 | 0.000 | 0.000 | 0.000 |  |
| Reliability | H | H | H | H | H | H |  |
|  | | | | | | | |
|  | **SGo/NGn (%)** | **L1NB（mm）** | **L1NB (°)** | **L1MP (°)** | **Y-axis (°)** | **Pog-NB (mm)** | **Pitch (°)** |
| ICC | 0.913 | 0.938 | 0.935 | 0.960 | 0.982 | 0.782 | 0.973 |
| 95%CI | 0.114~0.979 | 0.846~0.975 | 0.814~0.975 | 0.902~0.984 | 0.938~0.994 | 0.051~0.936 | 0.909~0.991 |
| p | 0.000 | 0.000 | 0.000 | 0.000 | 0.000 | 0.000 | 0.000 |
| Reliability | H | H | H | H | H | H | H |

H: ICC≥0.75; M: 0.4≤ICC<0.75; L: ICC<0.4

|  | **11H** | | | **11W-U1/3** | | **11W-L1/3** | | |
| --- | --- | --- | --- | --- | --- | --- | --- | --- |
| ICC | 0.987 | | | 0.983 | | 0.959 | | |
| 95%CI | 0.833~0.997 | | | 0.958~0.993 | | 0.895~0.984 | | |
| p | 0.000 | | | 0.000 | | 0.000 | | |
| Reliability | H | | | H | | H | | |
|  | | | | | | | | |
|  | **R23H** | **R23W-U1/3** | **R23W-L1/3** | | **L23H** | | **L23W-U1/3** | **L23W-L1/3** |
| ICC | 0.949 | 0.951 | 0.804 | | 0.903 | | 0.886 | 0.965 |
| 95%CI | 0.820~0.982 | 0.880~0.980 | 0.571~0.918 | | 0.761~0.961 | | 0.734~0.953 | 0.889~0.987 |
| p | 0.000 | 0.000 | 0.000 | | 0.000 | | 0.000 | 0.000 |
| Reliability | H | H | H | | H | | H | H |
|  | | | | | | | | |
|  | **R45H** | **R45W-U1/3** | **R45W-L1/3** | | **L45H** | | **L45W-U1/3** | **L45W-L1/3** |
| ICC | 0.965 | 0.879 | 0.944 | | 0.967 | | 0.922 | 0.965 |
| 95%CI | 0.913~0.986 | 0.717~0.951 | 0.867~0.977 | | 0.886~0.988 | | 0.814~0.968 | 0.915~0.986 |
| p | 0.000 | 0.000 | 0.000 | | 0.000 | | 0.000 | 0.000 |
| Reliability | H | H | H | | H | | H | H |
|  | | | | | | | | |
|  | **R67H** | **R67W-U1/3** | **R67W-L1/3** | | **L67H** | | **L67W-U1/3** | **L67W-L1/3** |
| ICC | 0.881 | 0.948 | 0.914 | | 0.944 | | 0.918 | 0.908 |
| 95%CI | 0.723~0.951 | 0.789~0.983 | 0.799~0.965 | | 0.797~0.981 | | 0.648~0.973 | 0.784~0.963 |
| p | 0.000 | 0.000 | 0.000 | | 0.000 | | 0.000 | 0.000 |
| Reliability | H | H | H | | H | | H | H |

H: ICC≥0.75; M: 0.4≤ICC<0.75; L: ICC<0.4

Supplemental Table B. Intra-observer correlation coefficient.

|  | **SNA (°)** | **SNB (°)** | **ANB (°)** | **Wits (mm)** | **MPSN (°)** | **FMA (°)** |  |
| --- | --- | --- | --- | --- | --- | --- | --- |
| ICC | 0.902 | 0.969 | 0.972 | 0.983 | 0.989 | 0.958 |  |
| 95%CI | 0.770~0.960 | 0.925~0.988 | 0.926-0.989 | 0.957-0.993 | 0.972-0.995 | 0.899-0.983 |  |
| p | 0.000 | 0.000 | 0.000 | 0.000 | 0.000 | 0.000 |  |
| Reliability | H | H | H | H | H | H |  |
|  | | | | | | | |
|  | **SGo/NGn (%)** | **L1NB（mm）** | **L1NB (°)** | **L1MP (°)** | **Y-axis (°)** | **Pog-NB (mm)** | **Pitch (°)** |
| ICC | 0.984 | 0.974 | 0.958 | 0.966 | 0.970 | 0.921 | 0.989 |
| 95%CI | 0.959-0.994 | 0.900-0.991 | 0.896-0.983 | 0.915-0.986 | 0.927-0.988 | 0.638-0.975 | 0.973-0.996 |
| p | 0.000 | 0.000 | 0.000 | 0.000 | 0.000 | 0.000 | 0.000 |
| Reliability | H | H | H | H | H | H | H |

H: ICC≥0.75; M: 0.4≤ICC<0.75; L: ICC<0.4

|  | **11H** | | | **11W-U1/3** | | **11W-L1/3** | |
| --- | --- | --- | --- | --- | --- | --- | --- |
| ICC | 0.994 | | | 0.979 | | 0.967 | |
| 95%CI | 0.984-0.998 | | | 0.947-0.991 | | 0.919-0.987 | |
| p | 0.000 | | | 0.000 | | 0.000 | |
| Reliability | H | | | H | | H | |
|  | | | | | | | |
|  | **R23H** | **R23W-U1/3** | **R23W-L1/3** | | **L23H** | **L23W-U1/3** | **L23W-L1/3** |
| ICC | 0.959 | 0.953 | 0.966 | | 0.978 | 0.911 | 0.969 |
| 95%CI | 0.900-0.983 | 0.886-0.981 | 0.917-0.986 | | 0.945-0.991 | 0.790-0.964 | 0.925-0.988 |
| p | 0.000 | 0.000 | 0.000 | | 0.000 | 0.000 | 0.000 |
| Reliability | H | H | H | | H | H | H |
|  | | | | | | | |
|  | **R45H** | **R45W-U1/3** | **R45W-L1/3** | | **L45H** | **L45W-U1/3** | **L45W-L1/3** |
| ICC | 0.838 | 0.886 | 0.949 | | 0.958 | 0.923 | 0.986 |
| 95%CI | 0.638-0.932 | 0.738-0.953 | 0.878-0.979 | | 0.896-0.983 | 0.819-0.969 | 0.966-0.995 |
| p | 0.000 | 0.000 | 0.000 | | 0.000 | 0.000 | 0.000 |
| Reliability | H | H | H | | H | H | H |
|  | | | | | | | |
|  | **R67H** | **R67W-U1/3** | **R67W-L1/3** | | **L67H** | **L67W-U1/3** | **L67W-L1/3** |
| ICC | 0.922 | 0.962 | 0.903 | | 0.933 | 0.981 | 0.960 |
| 95%CI | 0.814-0.968 | 0.909-0.985 | 0.771-0.961 | | 0.840-0.973 | 0.952-0.992 | 0.903-0.984 |
| p | 0.000 | 0.000 | 0.000 | | 0.000 | 0.000 | 0.000 |
| Reliability | H | H | H | | H | H | H |

H: ICC≥0.75; M: 0.4≤ICC<0.75; L: ICC<0.4
